# An ultrabrief, 2‐3 minute optimized cognitive biomarker for screening of MCI

**DOI:** 10.1002/alz70856_107266

**Published:** 2026-01-08

**Authors:** Daniel Z. Press, Maurice Smith

**Affiliations:** ^1^ Beth Israel Deaconess Medical Center, Boston, MA, USA; ^2^ Harvard University School of Enginering and Applied Sciences, Cambridge, MA, USA

## Abstract

**Background:**

Over 52 million people in the US are age 65 or over and at risk for Mild Cognitive Impairment, a condition affecting an estimated 12 million of them. With disease‐modifying therapies coming of age to slow the progression from MCI to dementia, identifying those with MCI is particularly critical as currently it is estimated that only 10% of those affected are diagnosed. An ultrabrief screening tool that could be performed in just 2‐3 minutes would empower primary care clinicians to improve routine screening for MCI.

**Method:**

We used a data‐driven approach to create a novel ultra‐brief tool, combining the most information‐efficient test‐items identified by analysis of NIH Uniform Data Set (UDS) version 3 study items in > 10,000 MCI and CN individuals from the National Alzheimer's Coordinating Center (NACC) data repository. In a manner similar to the development of the brief optimized cognitive composite (BOCC), here we created an ultra‐brief BOCC (uBOCC). This test does not require informant information and consists of just two optimally combined items (a 5‐word memory test and a category fluency test). We then measured the area under the receiver operating characteristics curve (AUC‐ROC) value for the uBOCC in detecting MCI.

**Result:**

The uBOCC showed an AUC‐ROC of 0.83 in predicting a diagnosis of MCI in the NACC. This accuracy is at least equal to the MoCA (0.82), while requiring only 2‐3 minutes. The test also performs exceptionally well at detecting future progression to dementia (a CDR‐Global score of 1 or greater) in 5 years with an AUC‐ROC of 0.91.

**Conclusion:**

An ultra‐brief screening tool, consisting of just two items optimally combined, can perform comparably to the MoCA and far outperform other brief screens such as the Mini‐Cog. Such a tool could be incorporated into routine screening in those over 65 to dramatically enhance MCI detection.

